# The risk factors of heart failure in elderly patients with hip fracture: what should we care

**DOI:** 10.1186/s12891-021-04686-8

**Published:** 2021-09-28

**Authors:** Fei You, Chaoyang Ma, Fangfang Sun, Lian Liu, Xiuwen Zhong

**Affiliations:** Department of Rehabilitation Medicine, Tongji Medical College, The Central Hospital of Wuhan, Huazhong University of Science and Technology, No.26 Shengli Street, Jiang’an District, Wuhan City, Hubei Province China

**Keywords:** Heart failure, Elderly, Hip fracture, Care, Management

## Abstract

**Background:**

Heart failure is a common adverse postoperative complication in elderly patients. It is necessary to explore the risk factors of heart after the operation of elderly patients with hip fracture during hospitalization.

**Methods:**

Patients with hip fractures admitted to our hospital from January 1, 2019 to December 31 2020 were included, all the patients received internal fixation surgery. The characteristics of patients with and without postoperative heart failure were compared. Multivariate logistic regression analyses were applied to analyze the risk factors of heart failure in elderly patients with hip fracture.

**Results:**

A total of 283 patients with hip fractures were included, the incidence of heart failure was 12.37 %. There were significant differences in the age, hypertension, anemia hypoalbuminemia and duration of surgery between heart failure and no heart failure group(all p < 0.05). There were no significant differences in the gender, BMI, diabetes mellitus, hyperlipidemia, history of heart failure, cognitive dysfunction, type of fracture, preoperative oxygen saturation, white blood cell count, platelet count, red blood cell count, creatinine, alanine aminotransferase, aspartate aminotransferase and estimated blood loss during surgery between heart failure and no heart failure group(all p > 0.05). Logistic regression analyses indicated that age ≥ 70y(OR2.446, 95% CI1.044 ~ 4.149), hypertension(OR2.152, 95% CI1.125 ~ 4.023), anemia(OR3.094, 95% CI1.294 ~ 5.907), hypoalbuminemia(OR2.377, 95% CI1.205 ~ 4.537), duration of surgery ≥ 120 min(OR1.683, 95% CI1.094 ~ 2.782) were the risk factors of heart failure in elderly patients with hip fracture(all *p* < 0.05).

**Conclusions:**

The incidence of postoperative heart failure in elderly patients with hip fracture is relatively high, which is the result of a combination of high-risk factors. Peri-period risk assessment and prevention of related risks are the keys to a good prognosis for patients.

## Background

Hip fracture is a common type of fracture in the elderly. It mainly refers to fractures of the proximal femur, including femoral neck fractures, intertrochanteric fractures and subtrochanteric fractures [1]. Femoral neck fractures and intertrochanteric fractures account for 90 % of hip fractures [2]. As the number of elderly people grows, by 2040, the number of hip fractures in the China will exceed 500,000 each year [3]. In general, the incidence of hip fractures increases with the age of patients [4]. Studies [5, 6] have found that the incidence of hip fractures in people older than 85 years is 10–15 times that of people aged 60–65. Due to the function decline of various organs in the body combined with other diseases and the influence of various complications in the elderly, the mortality after hip fracture is very high [7]. A large number of studies [8–10] have confirmed that compared with the population without fracture, patients with hip fracture have a significantly increased risk of death of 1.46 ~ 3.06 times. The high incidence of hip fractures in the elderly and the physiological characteristics of the elderly, and the presence of comorbidities and pathological conditions, specially geriatric syndromes, make the treatment of hip fractures in this specific population a major challenge for clinical orthopedic physicians [9, 11].

Studies [12, 13] have found that the occurrence of heart failure in elderly patients with hip fractures is common and serious. Heart failure after surgery can lead to delayed recovery, longer hospital stays, increased medical costs, and other complications such as bedsores, fall fractures, etc., which seriously affect the patient’s physical function and quality of life [14, 15]. Analyzing the exact high-risk factors that lead to postoperative heart failure is an effective way to clarify the mechanism, and can provide a theoretical basis for the prevention and treatment of postoperative heart failure. Therefore, this study aims to analyze the risk factors of heart failure after hip fracture in the elderly, to identify the potential influencing factors and possible mechanisms, and to provide a basis for the prevention and treatment of heart failure after surgery.

## Methods

### Ethics

This study was a retrospective cohort study. In this study, all methods were performed in accordance with the relevant guidelines and regulations. This present study was approved by the ethics committee of The Central Hospital of Wuhan(approval number: AC201802115), and informed consents had been obtained from all the included patients.

### Patients

We selected patients with hip fractures admitted to our hospital from January 1, 2019 to December 31, 2020, as the research objects. The inclusion criteria of the patients in this study were: age ≥ 60 years old; the patients had been diagnosed as hip fracture with no other fractures; patients received internal fixation surgery in our hospital; the patients were informed and willing to join this study. Exclusion criteria were: patients with incomplete clinical data; patients with heart failure before the surgery; patients with pathological fractures or advanced tumors; Patients with pulmonary embolism before surgery; Patients who were not willing to participate in this study.

### Heart failure diagnosis

Heart failure was diagnosed according to the “Guidelines for the Diagnosis and Treatment of Acute Heart Failure” formulated by the Cardiovascular Branch of the Chinese Medical Association, the heart failure was diagnosed based on clinical manifestations and auxiliary examinations [16]: The left ventricular ejection fraction (LVEF) decreased by < 45 %; there were signs of tachycardia, shortness of breath, lungs, pleural effusion, increased jugular vein pressure, peripheral edema, liver enlargement, heart chamber enlargement, third heart sound, heart murmur, abnormal echocardiogram, elevated levels of natriuretic peptide. We monitored the occurrence of heart failure after the operation immediately to the discharge of patients.

### Data collection

The two authors used a unified form to collect the patient’s personal characteristics and related treatment information, including age, body mass index (BMI), hypertension, diabetes mellitus, hyperlipidemia, history of heart failure, anemia, hypoalbuminemia, cognitive dysfunction, type of fracture, preoperative oxygen saturation, preoperative laboratory examination including white blood cell count, platelet count, red blood cell count, creatinine, alanine aminotransferase, aspartate aminotransferase, duration of surgery, estimated blood loss during surgery, methods of anesthesia including lumbar anesthesia and general anesthesia, estimated blood loss during surgery(blood loss = (weight of blood gauze-weight of dry gauze) + blood volume in suction bottle). Besides, we collected hemodynamic parameters during the surgery, including the average heart rate difference (ΔHR), systolic blood pressure difference (ΔSBP), diastolic pressure difference((ΔDBP), perfusion index (PI) difference (ΔPI), pulse oxygen perfusion variation index (PVI) difference (ΔPVI).

### Statistical Analysis

In this study, SPSS 25.0 software was used for statistical analysis. Measurement data were expressed as mean ± standard deviation, two independent sample t-tests were used for comparison between groups, count data were displayed as rates or percentages, and chi-square test was used for comparison between groups. Multivariate logistic regression model was used to analyze the risk factors of heart failure in elderly patients with hip fracture during hospitalization. In this study, P < 0.0 5 was regarded as the difference with statistical significance.

## Results

### The characteristics of included patients

A total of 283 patients with hip fractures were included in this present study, of whom 35 patietns had heart failure after surgery, the incidence of heart failure in elder patients with hip fracture was 12.37 %. As presented in Table 1, there were significant differences in the age, hypertension, anemia hypoalbuminemia and duration of surgery between heart failure and no heart failure group(all *p* < 0.05), and there were no significant differences in the gender, BMI, diabetes mellitus, hyperlipidemia, history of heart failure, cognitive dysfunction, type of fracture, preoperative oxygen saturation, white blood cell count, platelet count, red blood cell count, creatinine, alanine aminotransferase, aspartate aminotransferase and estimated blood loss during surgery, methods of anesthesia, ΔHR, ΔSBP, ΔDBP, ΔPI and ΔPVI(%) between heart failure and no heart failure group had been found(all *p* > 0.05).

**Table 1:** The characteristics of included patients

Variables	HF group(*n* = 35)	No HF group(*n* = 248)	t/χ^2^	p
Male/female	19/16	136/112	1.086	0.067
Age(y)	73.15 ± 8.92	66.93 ± 7.44	1.534	0.044
BMI (kg/m^2^)	22.63 ± 3.25	22.23 ± 2.09	4.131	0.071
Hypertension	25(71.43 %)	72(29.03 %)	1.094	0.008
Diabetes mellitus	11(31.43 %)	60(24.19 %)	1.282	0.057
Hyperlipidemia	9(25.71 %)	52(20.97 %)	1.699	0.102
History of HF	2(5.71 %)	12(4.84 %)	1.142	0.114
Anemia	19(54.29 %)	47(18.95 %)	1.422	0.036
Hypoalbuminemia	10(28.57 %)	34(13.71 %)	1.062	0.016
Cognitive dysfunction	3(8.57 %)	19(7.66 %)	0.966	0.087
Type of fracture			1.214	0.073
Intertrochanteric fracture	16(45.71 %)	110(44.35 %)		
Femoral neck fracture	19(54.29 %)	138(55.65 %)		
Preoperative oxygen saturation(%)	94.64 ± 10.31	95.75 ± 10.09	9.347	0.081
Preoperative laboratory examination				
White blood cell count (×10^9^ ·L^− 1^)	9.03 ± 1.61	8.93 ± 1.23	1.128	0.077
Platelet count (×10^9^ ·L^− 1^)	213.71 ± 84.52	210.24 ± 123.12	1.246	0.104
Red blood cell count(×10^9^ ·L^− 1^)	4.73 ± 1.17	4.66 ± 1.14	1.093	0.071
Creatinine (µmol·L^− 1^)	34.13 ± 14.36	33.26 ± 15.23	2.146	0.085
Alanine aminotransferase (U ·L^− 1^)	18.27 ± 10.12	17.13 ± 12.56	3.108	0.063
Aspartate aminotransferase(U ·L^− 1^)	19.34 ± 12.32	19.46 ± 15.30	2.997	0.102
Duration of surgery(min)	146.52 ± 66.49	112.59 ± 75.17	12.413	0.005
Methods of anesthesia			2.498	0.113
lumbar anesthesia	14(40.00 %)	105(42.34 %)		
General anesthesia	21(60.00 %)	143(57.66 %)		
Estimated blood loss during surgery(ml)	266.38 ± 44.52	260.14 ± 40.41	21.104	0.062
ΔHR(/min)	14.49 ± 3.12	15.18 ± 3.52	1.072	0.065
ΔSBP(mmHg)	17.62 ± 2.83	29.69 ± 4.55	5.128	0.092
ΔDBP(mmHg)	16.17 ± 2.24	15.52 ± 3.17	1.206	0.085
ΔPI(%)	3.18 ± 1.41	3.34 ± 1.28	1.299	0.106
ΔPVI(%)	3.96 ± 1.02	4.12 ± 1.21	1.384	0.059

### The risk factors of heart failure in elderly patients with hip fracture

We included the factors with significant difference in the univariate analysis for further multivariate logistic regression. The variable assignments of multivariate logistic regression were indicated in Table 2. As presented in Table 3, logistic regression analyses had found that age ≥ 70y(OR2.446, 95% CI1.044 ~ 4.149), hypertension(OR2.152, 95% CI1.125 ~ 4.023), anemia(OR3.094, 95% CI1.294 ~ 5.907), hypoalbuminemia(OR2.377, 95% CI1.205 ~ 4.537), duration of surgery ≥ 120 min(OR1.683, 95% CI1.094 ~ 2.782) were the risk factors of heart failure in elderly patients with hip fracture(all *p* < 0.05).

**Table 2:** The variable assignment of multivariate logistic regression

Factors	Variables	Assignment
HF	Y	Yes = 1, no = 2
Age(y)	X_1_	≥ 70 = 1, < 70 = 2
Hypertension	X_2_	Yes = 1, no = 2
Anemia	X_3_	Yes = 1, no = 2
Hypoalbuminemia	X_4_	Yes = 1, no = 2
Duration of surgery(min)	X_5_	≥ 120 = 1, < 120 = 2

**Table 3:** Logistic regression analysis on the risk factors of HF in elderly patients with hip fracture

Variables	β	S‾x	OR	95% CI	p
Age ≥ 70y	0.102	0.218	2.446	1.044 ~ 4.149	0.012
Hypertension	0.179	0.120	2.152	1.125 ~ 4.023	0.015
Anemia	0.132	0.137	3.094	1.294 ~ 5.907	0.036
Hypoalbuminemia	0.141	0.112	2.377	1.205 ~ 4.537	0.023
Duration of surgery ≥ 120 min	0.129	0.105	1.683	1.094 ~ 2.782	0.017

## Discussion

With the aging of the population and the development of modern medicine, the treatment mode of hip fracture has gradually evolved from “fracture” as the center to “elderly patients” as the center [17]. The organ function of elderly patients is gradually deteriorating. On the basis of chronic systemic diseases, the risks of surgery are naturally increased. Therefore, special attention should be paid to the perioperative management of elderly patients [18]. The aging of the population is a common challenge facing the world. Cardiovascular disease is the most common concomitant disease for elderly patients [19, 20]. Cardiovascular disease is closely related to cerebrovascular disease, diabetes, and kidney disease [21]. Hip fractures tend to occur in the elderly, and are often accompanied by various chronic diseases, poor organ compensatory function [22]. Several previous studies [23–25] have reported that hip fractures are closely associated with geriatric syndromes such as falls, frailty, sarcopenia, polypharmacy and degenerative neurological disorders. The surgical methods including hip replacement or internal fixation depend and are determined by the kind of fracture, and that trochanteric fractures are the most frequent fractures as the patient ages. Surgical treatment is the first choice, but the operation itself is also a kind of trauma, which may aggravate various existing medical complications, and the clinical symptoms of elderly patients with heart failure are not typical [26, 27]. The results of this study show that the incidence of postoperative heart failure in patients with hip fractures is relatively high, we have found that age ≥ 70y, hypertension, anemia, hypoalbuminemia, duration of surgery ≥ 120 min are the risk factors of heart failure in elderly patients with hip fracture. In clinical practice, medical staff should take early intervention for these risk factors to reduce the risk of postoperative complications.

Common surgical methods for hip fractures are hip joint replacement and internal fixation. Among them, hip replacement has the characteristics of large surgical trauma, longer operation time and high blood loss, and may also involve many problems such as bone cement implantation, while hip internal fixation has the advantages of relatively minimal invasiveness, short operation time and no bone cement implantation [28, 29]. In this study, only the impact of the difference between hip extramedullary fixation and intramedullary fixation on postoperative cardiovascular complications in elderly patients with hip fractures was discussed, and patients who underwent hip replacement were not included, which may weaken the influences of surgical approach [30]. A large number of studies [31–33] have focused on the cardiovascular events in patients undergone hip replacement, but very few focused on the patients undergone fixation treatment, our study findings, to some extent fill this gap.

Previous studies [34, 35] have shown that patients aged ≥ 70 years have a 7.9 % incidence of cardiovascular complications after knee arthroplasty. Therefore, for elderly patients, more detailed examination and evaluation should be performed before surgery. Anesthesiology consultation, optimization of internal medicine, attention to anesthesia management during the operation, reduction of surgical trauma and operation time, strict monitoring and multidisciplinary perioperative management after surgery [8, 36]. At the same time, it is also noted that even patients under 60 years of age, there will be serious cardiovascular complications after artificial joint replacement, the incidence of which is 2.7 %% [37]. For these non-high-risk patients, preoperative conversations should also explain the risk of cardiovascular events, and preoperative examination and perioperative management should not be ignored [38, 39].

Studies [40, 41] have shown that anemia is closely related to the prognosis of hip fractures. Studies have found that the mortality of patients with anemia at 3 and 12 months after discharge from the hospital is significantly higher than that of patients without anemia. At the same time, studies [42, 43] have shown that the mortality of patients with anemia has increased significantly. Anemia at admission for elderly patients with hip fractures is an independent risk factor for respiratory complications and fever, and an independent risk factor for postoperative complications in female patients [44]. This study also confirmed that anemia is an independent risk factor for postoperative heart failure. The possible mechanism is that the reduction of hemoglobin level can reduce the total amount of oxygen carried by red blood cells, and the hypoxia of body tissues promotes the release of nitric oxide, which leads to peripheral vasodilatation and decreased peripheral resistance, thereby activating the adrenal sympathetic nervous system and renin-angiotensin [45, 46]. The aldosterone system promotes the secretion of antidiuretic hormones, leading to water and sodium retention, which aggravates the symptoms of heart failure to a certain extent [47, 48].

The relationship between the length of surgery and postoperative heart failure is worth considering. This study found that the longer the operation time, the greater the risk of heart failure. It may be explained that the longer the operation time, the longer the patient’s stress time, resulting in greater stress on the heart [49]. In addition, patients often receive a certain amount of fluid infusion during surgery, the longer the duration of surgery, the more fluid will be infused, and the patient’s heart load will increase significantly [50]. The duration of hip fracture surgery is relatively long, and there are also certain differences in the duration of different surgical methods and materials [51, 52]. Therefore, clinical medical workers should focus on the appropriate surgical materials and methods before surgery, and at the same time strengthen the doctor’s surgical skills, shorten the operation time as much as possible, so as to reduce the incidence of heart failure in patients after the operation.

This study has the several shortcomings that must be concerned. Firstly, this study did not include patients with hip replacement, these patients are more prone to fluctuations in hemodynamics during the perioperative period, and the occurrence of postoperative cardiovascular events have been discussed in many previous published studies. We focused on patients undergoing internal fixation after hip fracture. Secondly, this study is a retrospective study. It lacks intraoperative hemodynamic indicators, postoperative pain treatment, and evaluation of patient subjective indicators, which remains to be further analyzed and discussed in follow-up studies. Besides, sodium retention is not only related to hypoaldosteronism stages in the elderly, but it is a more complex entity that involves physiological and pathological conditions, such as changes in body composition, ADH hypothalamic thresholds, inadequate pain control and other pathologies that leads to a syndrome of inappropriate secretion of antidiuretic hormone. Water and sodium retention is a vital factor that should be considered in the management of heart failure, we tried to collected more data about the water and sodium retention such as the fluid intake and serum sodium level to evaluate the associations of heart failure, but the relevant data were not complete. Thirdly, when hip fractures are clearly more related to female sex when we talk of frailty fractures among elderly population, yet our study had included more male patients than female patients, which might be associated with the population and area differences, and we only included two-years data in a single hospital. Future studies with larger sample size and multi-sites are needed.

## Conclusions

In conclusions, the postoperative heart failure is a common and serious complications for elder patients with hip fracture. The occurrence of postoperative heart failure of elderly hip fractures is the result of a combination of many factors, age ≥ 70y, hypertension, anemia, hypoalbuminemia, duration of surgery ≥ 120 min are the risk factors of heart failure in elderly patients with hip fracture. Early monitoring and correcting hypertension, anemia and hypoalbuminemia before surgery, shortening the duration of surgery are needed in clinical practice to reduce the occurrence of postoperative heart failure. Correct prediction and evaluation help patients to tide over the difficulties of surgery smoothly, minimize the risk of surgery, and enable patients to achieve the greatest degree of recovery.

## Data Availability

All data generated or analyzed during this study are included in this published article.

## Abbreviations

BMI: Body mass index
